# Deepening insights into cholinergic agents for intraocular pressure reduction: systems genetics, molecular modeling, and *in vivo* perspectives

**DOI:** 10.3389/fmolb.2024.1423351

**Published:** 2024-07-26

**Authors:** Minjae J. Kim, Mohamed M. Ibrahim, Monica M. Jablonski

**Affiliations:** ^1^ Department of Ophthalmology, The Hamilton Eye Institute, The University of Tennessee Health Science Center, Memphis, TN, United States; ^2^ Department of Pharmaceutics, Faculty of Pharmacy, Mansoura University, Mansoura, Egypt; ^3^ Department of Pharmaceutical Sciences, University of Tennessee Health Science Center, Memphis, TN, United States

**Keywords:** glaucoma, parasympathetic nervous system, systems genetics, virtual screening, molecular dynamics, quantitative structure-activity relationship, and *in vivo* efficacy

## Abstract

Parasympathetic activation in the anterior eye segment regulates various physiological functions. This process, mediated by muscarinic acetylcholine receptors, also impacts intraocular pressure (IOP) through the trabecular meshwork. While FDA-approved M3 muscarinic receptor (M3R) agonists exist for IOP reduction, their systemic cholinergic adverse effects pose limitations in clinical use. Therefore, advancing our understanding of the cholinergic system in the anterior segment of the eye is crucial for developing additional IOP-reducing agents with improved safety profiles. Systems genetics analyses were utilized to explore correlations between IOP and the five major muscarinic receptor subtypes. Molecular docking and dynamics simulations were applied to human M3R homology model using a comprehensive set of human M3R ligands and 1,667 FDA-approved or investigational drugs. Lead compounds from the modeling studies were then tested for their IOP-lowering abilities in mice. Systems genetics analyses unveiled positive correlations in mRNA expressions among the five major muscarinic receptor subtypes, with a negative correlation observed only in M3R with IOP. Through modeling studies, rivastigmine and edrophonium emerged as the most optimally suited cholinergic drugs for reducing IOP via a potentially distinct mechanism from pilocarpine or physostigmine. Subsequent animal studies confirmed comparable IOP reductions among rivastigmine, edrophonium, and pilocarpine, with longer durations of action for rivastigmine and edrophonium. Mild cholinergic adverse effects were observed with pilocarpine and rivastigmine but absent with edrophonium. These findings advance ocular therapeutics, suggesting a more nuanced role of the parasympathetic system in the anterior eye segment for reducing IOP than previously thought.

## 1 Introduction

**FIGURE 1:**
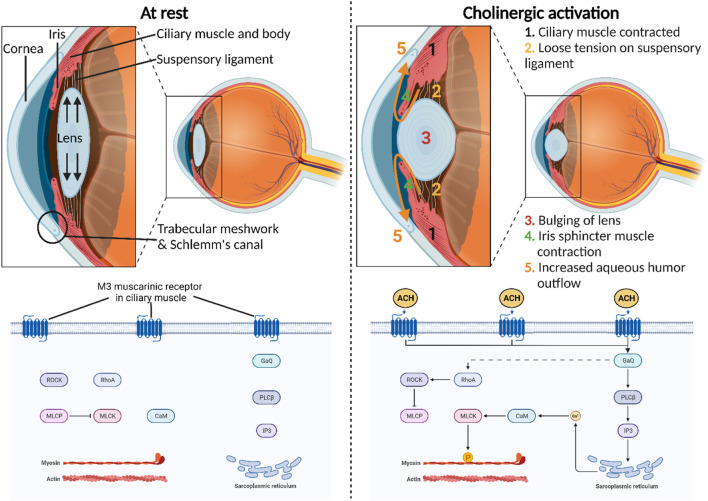
Physiological roles of cholinergic activation in the anterior segment of the eye. M3 muscarinic receptor is the most predominant subtype present in the human iris sphincter and ciliary body. The molecular mechanism of this receptor involves the direct stimulation of phospholipase C–β (PLCβ) and indirect stimulation of Ras homolog family member A (RhoA) through Gq proteins that ultimately release the inhibitory effect of myosin-light-phosphatase (MLCP) on myosin-light-chain kinase (MLCK). This intricate signaling cascade impacts calcium levels and subsequently MLCK to induce smooth muscle contraction.

Cholinergic activation assumes a central role in orchestrating diverse physiological functions within the anterior segment of the eye (Pakala et al., 2023). Triggered by the neurotransmitter acetylcholine release by postganglionic parasympathetic neurons, its interaction with muscarinic acetylcholine receptors occurs with exquisite precision and speed, modulating ocular dynamics within a matter of milliseconds (Vidal et al., 2023). Most notably, the binding of acetylcholine to the M3 muscarinic receptor (M3R) in the iris sphincter muscle enables its contraction, inducing pupil constriction (miosis) (Figure 1). This physiological response adapts the eye to varying light conditions, thereby enhancing visual acuity. Moreover, the activation of M3R plays a pivotal role in the contraction of the ciliary muscle, thereby governing two vital functions within the anterior segment of the eye. First, this contraction relaxes the tension in the suspensory ligaments linking the ciliary muscle to the lens, subsequently alleviating stretching tension within the lens (Wu et al., 2022). This, in turn, facilitates the accommodation of the lens, allowing for focused vision at different distances. Second, the contraction of the ciliary muscle exerts a tractional force on the trabecular meshwork, facilitating an increased outflow of aqueous humor through the conventional pathway (Keller and Peters, 2022). This regulatory mechanism provides an avenue for adjusting the intraocular pressure (IOP) in the eye, offering a potential target for innovative therapeutics designed to reduce IOP in glaucoma patients (Casson, 2022; Chen et al., 2023).

Muscarinic acetylcholine receptors comprise five distinct subtypes—M1, M2, M3, M4, and M5—each with specific roles and distributions in various tissues, including the eye (Thul and Lindskog, 2018; Ruan et al., 2021). These receptors operate through G protein-coupled receptor (GPCR) mechanisms, initiating intracellular signaling upon ligand binding (Kudlak and Tadi, 2023). M1 muscarinic receptors (M1R), primarily in the central nervous system, stimulate phospholipase C (PLC) via Gq proteins, affecting calcium levels and protein kinase C activation. M2 muscarinic receptors (M2R), prevalent in the heart, inhibit adenylate cyclase through Gi proteins, modulating cardiac activity and neurotransmitter release. M3Rs, which are widely distributed in smooth muscle and the eye, activate PLC through Gq proteins, influencing smooth muscle contraction and glandular secretion. M3Rs also indirectly activate Ras homolog family member A (RhoA) via Gq proteins, leading to the release of myosin-light-phosphatase (MLCP) inhibition on myosin-light-chain kinase (MLCK) (Balla et al., 2023). This complex signaling cascade influences calcium levels, ultimately affecting MLCK and inducing smooth muscle contraction. M4 muscarinic receptors (M4R), concentrated in the central nervous system, inhibit adenylate cyclase via Gi proteins, impacting neurotransmitter release. Lastly, M5 muscarinic receptors (M5R), found in various tissues, activate PLC through Gq proteins, influencing intracellular calcium levels and neuronal activity. Using radioligand binding, mRNA detection, and direct protein detection with monoclonal antibodies, research has unveiled the muscarinic receptor distribution in the human iris sphincter and ciliary body (Gil et al., 1997; Nietgen et al., 1999). This analysis highlights a prevalence of M3R, comprising approximately 60%–75% of the receptors. In contrast, lower proportions of M2R and M4R, ranging from 5% to 10%, are evident in these tissues. Notably, M1R, constituting 7%, is detected in the ciliary processes and iris sphincter. Curiously, M5R, which is typically localized to the central nervous system, was also detected in the iris sphincter, accounting for 5% of muscarinic receptors in this ocular region.

Primary open-angle glaucoma (POAG) is a leading global cause of blindness, characterized by progressive optic nerve damage and elevated IOP (Kwon et al., 2009). Steady-state IOP is generated by the balance of aqueous humor production by the ciliary body, drainage through the trabecular meshwork, and, to a lesser degree, the uveoscleral or nonconventional pathway (Llobet et al., 2003). The imbalance between aqueous humor production and drainage can lead to sustained pressure, compromising optic nerve health (Llobet et al., 2003; Tamm, 2009; Goel et al., 2010). Hence, therapeutic approaches targeting the predominant muscarinic receptor, M3R, have been developed to alleviate IOP in individuals with glaucoma. These strategies primarily involve two methods, both utilizing the endogenous ligand of muscarinic receptors, acetylcholine. The first method entails the direct activation of M3R using small molecules that mimic the chemical structure of acetylcholine, exemplified by pilocarpine—an FDA-approved M3R agonist—to reduce IOP (Chen et al., 2023). The second method involves inhibiting acetylcholine degradation at the synapse between the axon terminal of post-ganglionic parasympathetic neurons and cholinergic targets in the eye. This is achieved using small molecule acetylcholinesterase inhibitors, such as physostigmine, to transiently elevate acetylcholine levels and hyper-activate M3R, thereby lowering IOP (Andrade and Zafar Gondal, 2023). Despite their proven efficacy in lowering IOP, both pilocarpine and physostigmine exhibit substantial adverse effects that constrain their clinical application. These drugs are associated with potent systemic cholinergic side effects, including excessive sweating, lacrimation, urinary incontinence, diarrhea, muscle weakness, airway constriction, and cardiac arrhythmia (Arens and Kearney, 2019; Panarese and Moshirfar, 2023). Consequently, cholinergic drugs have largely been supplanted by alternative IOP-reducing medications with distinct mechanisms, such as beta-blockers, prostaglandin analogs, and rho-kinase inhibitors (Liu et al., 2022). These alternatives avoid the issues of accommodation and pupillary reflex loss associated with cholinergic drugs. Nonetheless, these medications still present drawbacks that curtail their clinical utility. Beta-blocker IOP drops, for instance, have been linked to adverse effects, like headaches, dizziness, impotence, and other discomforts in glaucoma patients (Haga et al., 2022). In addition, FDA-approved prostaglandin analogs, like Vyzulta (latanoprostene bunod, 0.024%), Travatan Z (travoprost,0.004%), and Lumigan (bimatoprost, 0.03%), may cause permanent pigmentation of the eyelids, lashes, and iris (Llobet et al., 2003; Beidoe and Mousa, 2012; Shah et al., 2013). Lastly, while Rho kinase inhibitors are generally well-tolerated, they can induce conjunctival hyperemia, instillation site pain, and conjunctival hemorrhages, underscoring the imperative for safer drug options (Moshirfar et al., 2018). Hence, in this investigation, we revisit the parasympathetic nervous system within the anterior segment of the eye with the objective of repurposing existing FDA-approved or investigational drugs that selectively target this system to reduce IOP while minimizing potential side effects. This led our team to first conduct systems genetics analyses and molecular modeling to screen for potential lead compounds capable of IOP reduction. Subsequently, we employed mouse models to assess their efficacy in lowering IOP and to screen for potential cholinergic adverse effects.

## 2 Materials and methods

### 2.1 Correlation analyses of muscarinic acetylcholine receptor genes and IOP

Systems genetics is a methodology that investigates the genetic basis of disease traits by simultaneously analyzing extensive sets of genotypes and phenotypes (Andreux et al., 2012; Houtkooper et al., 2013; Wang et al., 2016). The BXD family, a well-characterized mouse genetic reference population, combines C57BL/6J and DBA/2J mice genomes via spontaneous recombination over 20+ generations of inbreeding, segregating for approximately 5.5 million sequence variants (Taylor et al., 1999). This family of experimental mice has been genotyped and phenotyped thoroughly at many levels, from messenger RNA levels to IOP (Chesler et al., 2005; Ashbrook et al., 2015; Williams et al., 2016). This makes the BXD family a valuable resource for glaucoma-related systems genetics studies. First, transcript data for *Chrm1, Chrm2, Chrm3, Chrm4,* and *Chrm5* (corresponding to M1R, M2R, M3R, M4R, and M5R, respectively) were acquired from Gene Network 2.0 (https://genenetwork.org/) (Mulligan et al., 2017). The comprehensive transcript data are openly accessible at UTHSC BXD All Ages Eye RNA-Seq (Nov20) TPM Log2 - eye mRNA database. Similarly, IOP measurements from 51 strains of BXD mice of all ages (1–30 months old), both sexes, average of left and right eyes were obtained, which are openly accessible at BXD Published Phenotypes–IOP database. Pearson correlation matrix analysis (*p*-value <0.05) was then conducted, following the instructions in the user’s manual at Gene Network 2.0, to identify statistically significant correlations between IOP and the five genes of interest. Lastly, another round of analysis was performed to identify statistically significant correlations among the mRNA expressions of the five genes.

### 2.2 Homology modeling of human M3R

In drug discovery, molecular modeling is commonly used to visualize the binding interaction of a ligand to a known protein structure (Kim et al., 2023). The ligand is docked against a specific site on the protein of interest, and the best binding and docking score are calculated based on the energy reduction of the two entities before and after the docking. A more negative docking score means a higher energy reduction and thus a tighter binding between the ligand and the protein (Guedes et al., 2014). Ideally, molecular modeling is most effectively performed using a protein structure established through X-ray crystallography, electron microscopy, or nuclear magnetic resonance imaging, and published in the Protein Data Bank for reference. Within the protein data bank, there were a total of ten published structures corresponding to the entry name “Muscarinic acetylcholine receptor M3,” composed of four human M3R and six rat M3R structures. All the human M3R structures, however, underwent significant modifications, like mutagenesis, truncation, separation, and chemogenetic alterations, that limited their utilities for molecular modelling (Zhang et al., 2022). Hence, homology modelling was pursued to predict the full structure of the human M3R before proceeding with the docking analysis. Homology modeling is considered the most accurate among the computational structure prediction methods in this situation (Muhammed and Aki-Yalcin, 2019). The full amino acid sequence of human M3R was first acquired from UNIPROT database, and its molecular weight as well as isoelectric point were predicted based on its amino acid sequence using SWISS-EXPASY MW/pI calculator (Audain et al., 2016) (Supplementary Figure S1). Human M3R structure was constructed via homology modeling using Sequence viewer function in Maestro (Schrödinger, Inc.) with a partially solved structure of human M3R in complex with iperexo, a muscarinic receptor super-agonist, (PDB: 8E9Z) as template structure with sequence identity of 100%. It is essential to note that our homology model incorporates a truncating modification in intracellular loop three spanning amino acid positions from 253 to 491, a common practice for membrane proteins with complex intracellular loops to enhance stability prior to MD simulation (Li et al., 2024; Stampelou et al., 2024). However, given that our study focuses on the transcellular binding pocket of M3R, we anticipate that the altered intracellular loops will not impact this interaction. Subsequently, assignment of bond orders and hydrogenation for the complex structures were conducted using Protein Preparation in Maestro. The ionization state of the human M3R suitable for pH 7.0 ± 2.0 was predicted using Epik (Shelley et al., 2007). H-bond optimization was conducted using PROPKA (Li et al., 2005). Energy minimization was conducted utilizing the OPLS3e force field to release the protein conformation from its initially crystallized state of M3R agonism associated with the template structure (Roos et al., 2019). Subsequently, loop structures were refined using Loop Refinement feature in Maestro. Lastly, a quantitative structural analysis of the homology model was performed on both the template structure and homology model using Alignment root-mean-square-deviation (RMSD) and Ramachandran plot analysis in Maestro. RMSD calculates the difference in atomic distance between template structure and homology model, where lower the RMSD the closer the homology model is to the template structure in 3D conformation. Each dot on the Ramachandran plot represents a peptide with corresponding phi-psi angles. Blue dots indicate “allowable” peptides with favorable phi-psi angles, while red dots indicate “disallowed” peptides due to unfavorable phi-psi angles. Peptides with phi/psi angles around −90/120 degrees correspond to beta sheets, −90/-30 degrees to right-handed alpha helices, and 90/60 degrees to left-handed alpha helices.

### 2.3 Molecular docking of compound libraries against human M3R

First, the binding site of the human M3R homology model was determined based on relevant literature sources (Martinez-Archundia et al., 2012; Kistemaker et al., 2019). Modified protocols from previous modelling studies and relevant literature were adopted for molecular docking (Saini et al., 2019; Li et al., 2021). The identified binding residues were subsequently utilized as input for the Receptor Grid Generation function in Maestro, creating a three-dimensional exploration space for molecular docking. The dimensions and center point coordinates of the grid box were set to 20 Å × 20 Å × 20 Å and 52.34, 95.03, 59.03 as coordinates for *X*, *Y*, *Z*-axis of the center point, respectively. Receptor grids were generated, and various classes of ligands were prepared for docking using the LigPrep function in Maestro (Wishart et al., 2018). First, a compound library was acquired from the DrugBank 6.0 database, consisting of six known agonists of M3R to serve as positive controls (pilocarpine, NGX267, bethanechol, cevimeline, xanomeline, methacholine) and 15 known antagonists of M3R to serve as negative controls (tiotropium, diphenidol, tridihexethyl, methanetheline, solifenacin, glycopyrronium, oxybutynin, oxyphencyclimine, promethazine, homatropine methylbromide, procyclidine, dicyclomine, olanzapine, ipratropium, tropicamide) for our docking study (Vacca et al., 1987; Greig et al., 2013; Knox et al., 2024). Following ligand preparation, the receptor grid files were employed in the Glide function, with extra-precision selected as the screening method and “amide-only” chosen for interaction settings. The torsional degrees of freedom of the ligand molecule were determined by its flexibility. Structural motifs associated with binding interactions were computed and generated using the “Ligand Interaction Diagram” software installed within Maestro interface (Basaran et al., 2024). This tool employs a sophisticated array of computational techniques to predict and visualize key interactions between proteins and ligands, including hydrogen bonds, van der Waals forces, covalent, and ionic bonds. The “Ligand Interaction Diagram” tool visualizes the results of Glide dock, which uses geometric criteria to identify hydrogen bonds, evaluating optimal distances and angles between donor and acceptor atoms, which are visualized as dashed lines in accompanying tools. Van der Waals interactions are modeled using Lennard-Jones potentials, highlighting favorable contacts through molecular surface displays or depicting close atomic contacts. While covalent bonding is generally predefined in the molecular structures, its visualization is managed through representations showing bonds as sticks or lines. Ionic bonds are detected through assessments of electrostatic potentials between charged groups, with visual representations often differentiated from hydrogen bonds by a mix of red and blue colors. This suite of computational predictions allows integrative assessment of various structural motifs predicted by Glide docking.

After completing the docking for positive and negative controls, a compound library consisting of 1,667 FDA-approved or investigational drugs was acquired from ZINC 5.0 database and prepared for docking as previously described. Due to the large size of the ligand sample, a series of molecular docking procedures was performed. Each successive series was conducted with higher precision but longer computational time. Glide docking operates at three precision levels: High Throughput Virtual Screening (HTVS), Standard Precision (SP), and Extra Precision (XP). HTVS is a less stringent method that discards ligands if their volume exceeds that of the binding site. SP is more computationally intensive, providing more accurate predictions by employing more thorough sampling and refinement processes. XP, on the other hand, performs even more extensive sampling than SP and uses a sophisticated scoring function that demands stricter ligand-receptor shape complementarity, thereby reducing false positives. XP also evaluates ligand fit to specific receptor conformations, which is useful for proteins with conformational flexibility. Within the context of our study, a compound library of 1,667 compounds underwent docking via HTVS mode with “amide-only” chosen for interaction settings, initially reducing the number of ligands to 82 compounds. Hit compounds generated underwent a secondary docking process using the SP mode in Glide for further refinement of the drug library, further reducing the library to 23 compounds. Finally, the docked ligands underwent a conclusive, third docking process using XP mode in Glide to complete the refinement to four compounds. These finalized hit compounds were then categorized as potential M3R agonists or antagonists based on the structural motifs identified from the initial docking process using the six positive and 15 negative controls. All four compounds housed a positively charged tertiary nitrogen, facilitating hydrogen and ionic bonding with M3R binding residues—a characteristic consistently observed across all 21 controls. Intriguingly, two of the compounds, sulpiride and profenamine, exhibited π-π stacking interactions with a tryptophan residue in M3R, a feature predominantly associated with 12 of the 15 M3R antagonists examined. Conversely, the other two compounds, rivastigmine and edrophonium, did not display this interaction, aligning instead with the structural themes of the agonists. To further explore the dynamic interactions and stability of these structural motifs, our team initiated MD simulations extending over 100 nanoseconds using pilocarpine and tiotropium, as known M3R agonist and antagonist controls, respectively.

### 2.4 Molecular dynamics (MD) simulation of human M3R in complex with pilocarpine and tiotropium

Utilizing Desmond (Schrödinger Inc., USA), MD simulations were executed to explore interactions and calculate binding free energy, aiming to elucidate the differences in binding modes for M3R agonism and antagonism. Modified protocols from previous studies and relevant literature were adopted (Saini et al., 2019; Kim et al., 2023). The binding sites of pilocarpine and tiotropium were verified according to the literature (Kruse et al., 2012). Configuration of all systems was performed using the “System Builder” tool in Maestro. The protein-ligand complex was positioned in an orthorhombic box with a 10 Å buffer distance, incorporating a hydration model with the SCP water model that was equilibrated with salt concentrations of 0.15 M Na^+^ and 0.15 M Cl^−^ at physiological pH. Studies with similar MD simulation workflow as our study reported that both SPC and TIP4P (Transferable Intermolecular Potential 4 Point) water models reproduce the best experimental values (Zielkiewicz, 2005). Choosing the SPC water model over the TIP4P model was based on considerations of computational efficiency and compatibility with other simulation components. SCP models, including variations like SPC/E, are less computationally demanding due to their simpler three-point charge system, which excludes the additional interaction site found in TIP4P. This makes SCP models faster, which is advantageous for large systems or extensive simulations (Steinberg et al., 2019). While TIP4P may offer superior accuracy for certain thermodynamic properties, SCP models still provide reasonable approximations for many applications where ultra-precise water behavior is not critical. Furthermore, the SCP model’s compatibility with widely used force fields and its straightforward implementation make it a practical choice, especially for those new to molecular dynamics or when working under computational constraints. This balance of efficiency, sufficient accuracy, and ease of use led our team to choose SCP over TIP4P.

Subsequently, key parameters, including van der Waals cut-off radius, time step, initial temperature, and system pressure, were set to 9 Å, 2.0 fs, 300 K, and 1.01325 bar, respectively. While the size of the receptor itself measured at approximately 11,000 atoms, the size of the system containing the protein-ligand complex with the buffer measured at approximately 36,000 atoms and total volume of 628.84 Å^3^. MD simulations, lasting 100 ns under the NPT ensemble, were executed with a simulation sampling interval of 100 ps. A simulation time of 100 ns was selected for this study because pilocarpine and tiotropium are both known ligands of M3R. Our team determined that this duration would allow sufficient time for the protein and ligand to establish stable interactions. This choice aligns with other recent studies using molecular dynamics to simulate and analyze known interactions in real-time under physiological conditions (Gheidari et al., 2024; Roy et al., 2024). Subsequently, trajectories from each MD simulation were scrutinized using the Simulation Interactions Diagram tool in Maestro. Trajectory Frame Clustering in Maestro determined the most populated representative structure for each MD simulation before conducting molecular mechanics with generalized Born and surface area solvation (MM/GBSA) analysis for free energy calculation using Prime (Schrödinger Inc., USA). This entire procedure, from system configuration to MM/GBSA analysis, was performed three times for each protein-ligand complex. While the trajectory analysis yielded identical results across all replicates for each complex, we observed slight variations in the MM/GBSA analysis.

### 2.5 Molecular mechanics with generalized born and surface area solvation (MM/GBSA) analysis for free energy calculation

In MD simulation, free energy calculations provide quantitative production of protein–ligand binding energies. The binding energy (ΔG_bind_) is calculated by Eq. 1:

$$∆G_{\mathrm{bind}}=G_{R+L}-\left(G_{R}+G_{L}\right)$$ (1)

where $G_{R+L}$ represents the hM3R in complex with ligands, while $G_{R}$ and $G_{L}$ represent the optimized free hM3R and optimized free ligand Gibbs energies, respectively (Froloff et al., 1997).

In the MM/GBSA approach (Genheden and Ryde, 2015), each free energy term in Eq. 1 is calculated using Eq. 2.

$$G=G_{\mathrm{coulomb}}+G_{\mathrm{vDW}}+G_{\mathrm{covalent}}+G_{\mathrm{solv}}+G_{\mathrm{self}‐\mathrm{contact}}+G_{H‐\mathrm{bond}}+G_{\mathrm{lipo}}+G_{\mathrm{packing}}$$ (2)

where $G_{coulomb}$ represents Coulombic energy, $G_{vDW}$ Van der Waals energy, $G_{covalent}$ covalent binding energy, $G_{solv}$ Generalized Born electrostatic solvation energy, $G_{self-contact}$ self-contact energy, $G_{H-bond}$ hydrogen-bonding energy, $G_{lipo}$ lipophilic energy, and $G_{packing}$ pi-pi packing energy.

The performance of the MM/GBSA algorithm is based on the specificity of the forcefield and ligand partial charges, the specificity of protein–inhibitor complex, MD simulation, inner dielectric constant, and the docking pose number based on top scoring (Halim et al., 2021). The VSGB solvation model (Li et al., 2011) and OPLS3e force field were set for the calculation via MM/GBSA feature in Maestro.

### 2.6 Assessment of lead compounds for IOP reduction in mice

A total of six C57BL/6 J mice (n = 6; 3 males and 3 females, aged 2–6 months) were employed to assess the IOP-lowering effects of lead compounds identified through molecular docking and molecular dynamics simulations. As the drug molecules were soluble in aqueous media, they were easily prepared as aqueous solutions. However, to prevent rapid drainage from the eye surface before eliciting a pharmacological response, these molecules were incorporated into bioadhesive viscous Carbopol 981 eye drops. A 0.4% weight/volume (w/v) concentration of Carbopol 981 was soaked in Milli-Q water overnight for full swelling. Equal volumes of 0.4% w/v Carbopol 981 gel and drug aqueous solutions were mixed using a vortex mixer to create the final bioadhesive viscous eye drops, stored in closed, air-tight containers at 5 °C in darkness until use. The resulting products contained 0.6% w/v of each drug in 0.2% w/v Carbopol 981 gel. To ensure sterility, all tools and water used were sterile, and the procedures were carried out under aseptic conditions.

For each mouse, the tested ophthalmic formulation (10 μL) was applied to both right and left eyes. IOP of mice was subsequently measured every hour for the next 8 h using an induction-impact tonometer (Tonolab tonometer, Colonial Medical Supply, Franconia, NH) according to the manufacturer’s recommended procedures. Six consecutive IOP readings were averaged. All results were expressed as the mean percentage reduction in IOP from baseline (mean % reduction ± SEM). All procedures involving mice were approved by the Animal Care and Use review board of the University of Tennessee Health Science Center (UTHSC) and followed the Association of Research in Vision and Ophthalmology Statement for the Use of Animals in Ophthalmic and Vision Research in addition to the guidelines for laboratory animal experiments (Institute of Laboratory Animal Resources, Public Health Service Policy on Humane Care and Use of Laboratory Animals). Animals were housed under cyclic light (12 h on:12 h off) with 35% humidity in a specific pathogen-free facility at UTHSC and were allowed free access to water and food.

### 2.7 Quantitative structure-activity relationship (QSAR) and ADME (absorption, distribution, metabolism, excretion) analysis of cholinergic drugs

The 3D structures of pilocarpine, physostigmine, rivastigmine, edrophonium, sulpiride, and atropine were subjected to QSAR analysis using physics-based membrane permeability tool in Maestro (Leung et al., 2012; Leung et al., 2016). The physiochemical properties derived mathematically from QSAR were then used to predict the corneal permeability of the investigated cholinergic drugs (Karami et al., 2022). To calculate changes in free energy of partitioning (ΔG_o/w_), we first obtained the distribution coefficient (cLogD at pH 7.4) and partition coefficient values (cLogP). The free energy of distribution value was then calculated based on mathematical equations adapted from the literature (Anderson et al., 1988; Leung et al., 2012; Karami et al., 2022). In the QSAR analysis, cLogD_pH7.4_ represents the distribution coefficient for a drug at pH 7.4, which is the pH of the tear film. The higher the cLogD_pH7.4_, the higher the hydrophobicity of the drug. ΔG_o/w_ represents the change in Gibbs free energy of distribution/partitioning of a drug, where higher the value the higher the hydrophilicity. Finally, TPSA represents the topological polar surface area of a drug, where the higher the TPSA the greater the physiochemical space occupied by the polar surface of the drug. The rule of thumb for optimal ocular permeability and absorption for topical eye drops proposed by Karami et al. (RO_x_) suggests that drugs with cLogD_pH7.4_ ≤ 4.0, ΔG_o/w_ ≤ 20 kcal/mol, and TPSA ≤ 250 Å are in favor of crossing the corneal membrane upon topical delivery (Karami et al., 2022). Lastly, the SDF files of pilocarpine, physostigmine, rivastigmine, edrophonium, sulpiride, and atropine were uploaded to SWISS-ADME, a freely accessible web tool for evaluating pharmacokinetics, drug-likeness, and medicinal chemistry properties of small molecules (Daina et al., 2017). Specifically, LogP represents the partition coefficient, indicating the compound’s lipophilicity and ability to cross the cell membrane, while LogS denotes its aqueous solubility, impacting absorption and bioavailability. GI absorption assesses the likelihood of a compound being absorbed through the gastrointestinal tract. CYP interactions highlight the compound’s interactions with cytochrome P450 enzymes, which are crucial for its degradation and metabolism. BBB (blood-brain barrier) permeability measures the compound’s ability to cross the blood-brain barrier, and P-gp substrate status identifies whether the compound is a substrate for P-glycoprotein, a transporter protein that can affect its absorption and excretion. These data collectively aimed to enhance our understanding of the pharmacokinetics of the cholinergic drugs in relation to their observed biological activities in mice.

### 2.8 Data and statistical analysis

The data and statistical analysis in this study comply with the recommendations of the British Journal of Pharmacology on experimental design and analysis in pharmacology. A change is considered statistically significant, if the statistical analysis yields a *p-*value <0.05. Pearson product-moment correlations were computed using the Correlation matrix tool within GeneNetwork 2.0. Denoted by “r,” the Pearson correlation assesses the strength of a linear association between two variables, specifically in our case, IOP (mmHg) and muscarinic acetylcholine receptor mRNA expressions. The calculated pharmacodynamic parameters for the lead compound encompass maximum percent IOP reduction, the time to reach the maximum decrease in percent IOP (T_max_), the duration for IOP to return to baseline (i.e., end of drug effect; T_end_), and the total area under the percent IOP reduction-versus-time curve (AUC). GraphPad Prism-10 software (GraphPad Software Inc., San Diego, CA, USA) was employed for all pharmacodynamic calculations, and the results were presented as the mean ± SEM.

### 2.9 Materials

All modelling operations in Maestro were performed using RedHat Enterprise Linux (RHEL) Version 8.4 Desktop computer containing two Linux X64 (AMD64/EM64T) Display GPU Drivers. Tested compounds, namely pilocarpine, rivastigmine, edrophonium, sulpiride, and atropine, were purchased from Selleckchem (New York, United States) with catalog numbers of S4231, S4713, S2087, S5900, S4655, and S4713, respectively. Meanwhile, our last tested compound, physostigmine, was purchased from MedChemExpress (New Jersey, United States) with a catalog number of HY-B1266.

## 3 Results

### 3.1 Correlation analyses of muscarinic acetylcholine receptor genes and IOP

**FIGURE 2:**
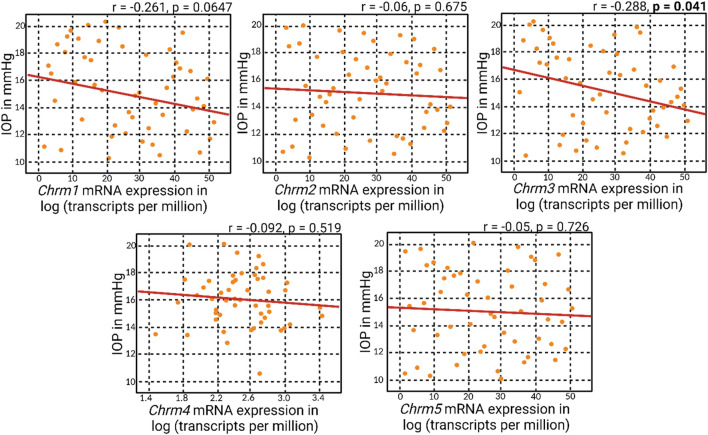
Correlation analyses of muscarinic acetylcholine receptor genes and IOP. Correlation matrix analyses revealed an overall negative correlation between muscarinic acetylcholine receptor mRNA expression and IOP. Notably, the statistically significant negative correlation was observed only with *Chrm3* mRNA expression, corresponding to M3R.

Correlation matrix analyses revealed an overall negative correlation pattern between muscarinic receptor mRNA expression and IOP (Figure 2). However, the negative correlation attained statistical significance solely with *Chrm3* mRNA expression, corresponding to M3R. This particular outcome aligns with the understanding that M3R stands out as the predominant muscarinic receptor subtype within the anterior segment of the eye. The specificity of the negative correlation with *Chrm3* mRNA also reinforces the notion that the influence of muscarinic receptors on IOP is particularly associated with the M3 subtype, substantiating its pivotal role in modulating IOP dynamics.

**FIGURE 3:**
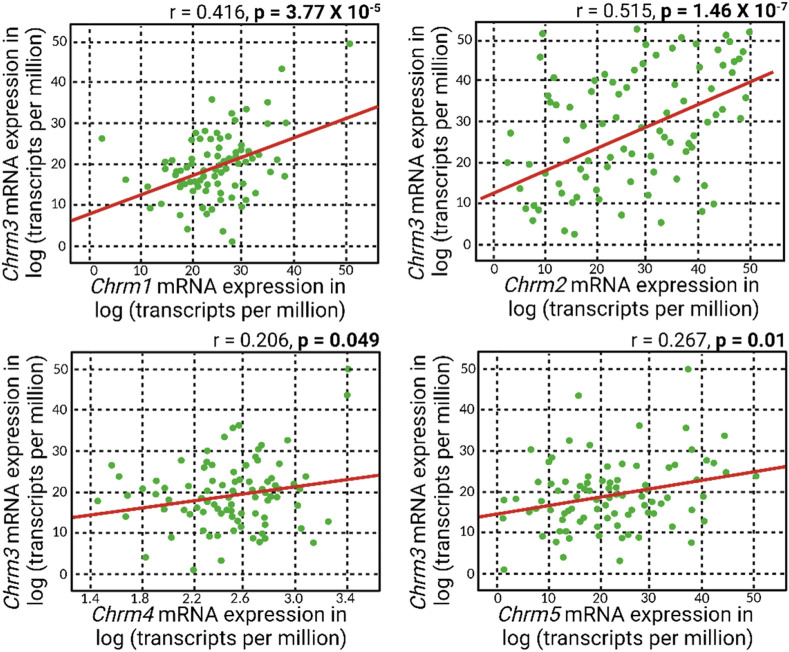
A positive correlation pattern manifested in the association between *Chrm3* and the mRNA expressions of other muscarinic acetylcholine receptors. Remarkably, all these positive correlations attained statistical significance, and the most robust correlation was evident between *Chrm2* and *Chrm3* mRNA expressions.

Meanwhile, a general positive correlation pattern emerged between *Chrm3* and the mRNA expressions of other muscarinic acetylcholine receptor subtypes (Figure 3). Notably, all these positive correlations reached statistical significance, with the most robust correlation observed between *Chrm2* and *Chrm3* mRNA expressions. This finding suggests a potential regulatory role of M3R expression on other muscarinic subtypes, indicating a coordinated and interrelated regulation of their respective expressions. The intricate interplay among these receptors could play a role in modulating IOP, given all five muscarinic acetylcholine receptors are expressed in the anterior segment of the eye.

### 3.2 Human M3R homology model and its binding site

**FIGURE 4:**
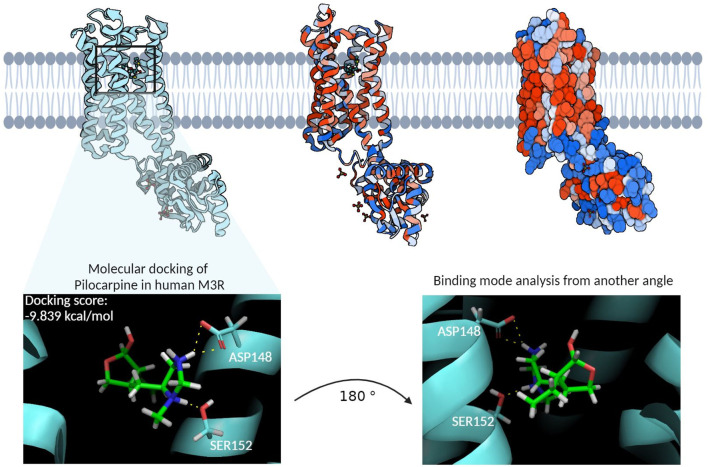
The homology model of the human M3R and its binding site. The cyan structure on the left represents the ribbon diagram of human M3R, while the middle structure provides a van der Waals ribbon diagram, where red regions denote hydrophobic residues and blue regions hydrophilic residues. The right structure illustrates the contact surface diagram of human M3R, with red regions corresponding to hydrophobic residues and blue regions to hydrophilic residues. The molecular docking of pilocarpine, a recognized agonist, into this binding site resulted in a docking score of −9.839 kcal/mol. Particularly noteworthy in the interaction profile were the extensive hydrogen bonding interactions involving pilocarpine’s positively charged tertiary amine group and the polar structures of ASP148 and SER152. This structure subsequently underwent MD simulations to identify additional binding residues that may enhance the stability of this interaction.

The homology model of the human M3R housed its binding site within the hydrophobic core of its transmembrane domain (Figure 4). Molecular docking of pilocarpine, a known agonist, into this binding site revealed a docking score of roughly −10 kcal/mol. Particularly noteworthy in the binding mode were the hydrogen bond interactions from ASP148 and SER152, respectively, involving pilocarpine’s hydrogen atoms associated with its tertiary amine group. This is consistent with the binding mode of iperexo, an M3R super-agonist, in our template structure (PDB:8E9Z), in which the ligand’s tertiary amine group is seen associating with ASP148, SER152, and TYR530 via cation-π and hydrogen bond networks (Supplementary Figure S2 (Zhang et al., 2022). Both iperexo and pilocarpine displayed similar 3D conformations within the M3R binding site, with their positively charged tertiary amine oriented towards ASP148 and their aromatic rings towards VAL156. Furthermore, the binding modes of both compounds involved extensive π-cation and salt bridge interactions with the nitrogen ring via ASP148, TYR149, and TYR507, while there was no evidence of π-π stacking interaction with TRP504 in both compounds. Finally, pilocarpine exhibited a unique hydrogen bonding interaction between its carbonyl ring and TRP200.

Structural alignment of our homology model to the template structure revealed an all-atom RMSD of 4.283 Å and a central carbon (Cα) backbone RMSD of 0.005 Å. This discrepancy between the all-atom and Cα RMSD indicates that most of the variations in the peptide structures arise from the flexibility of the amino acid side chains. While an all-atom RMSD of less than 2.0 Å is ideal for homology models, an RMSD of less than 5.0 Å is also considered reasonably appropriate (Zhang and Skolnick, 2004; Kumar et al., 2019). Subsequent Ramachandran plot analysis compared the structural motif of our template structure (PDB: 8E9Z) to that of our homology model (Supplementary Figure S3. Overall, similar distributions of peptides were observed for all secondary structures, specifically beta-sheets and alpha-helices. However, our homology model displayed 30″disallowed” peptides, as opposed to the template model’s two “disallowed” peptides. In terms of structural quality of our homology model, an all-atom RMSD of 4.283 Å and a percentage “disallowed” peptides of 8.5% (30/352) suggest that there is still room for improvement in the quality of our homology model. This higher number of “disallowed” regions in the Ramachandran plot may be attributed to the methodology employed for constructing the model in Maestro. Specifically, the approach of attempting to model the entire protein in a single, unified step, rather than adopting a systematic, domain-wise modeling strategy followed by a careful integration of these domains using appropriate constraints, could have led to inaccuracies. Such a domain-wise approach often enables more precise control over the conformational space of each domain, potentially reducing the occurrence of structurally implausible regions (Xia et al., 2023). This underscores the need for refining our modeling strategies to enhance the structural accuracy of complex protein models. However, the residues within the canonical binding site of M3R, namely ASP148, TYR149, SER152, and TYR530, all had favorable phi-psi angles. Given that the focus of our study is within the transmembrane domain rather than looped domains with flexible amino acid side chains, our team proceeded with molecular docking and MD simulations.

### 3.3 Molecular dynamics simulation of human M3R in complex with pilocarpine and tiotropium

**FIGURE 5:**
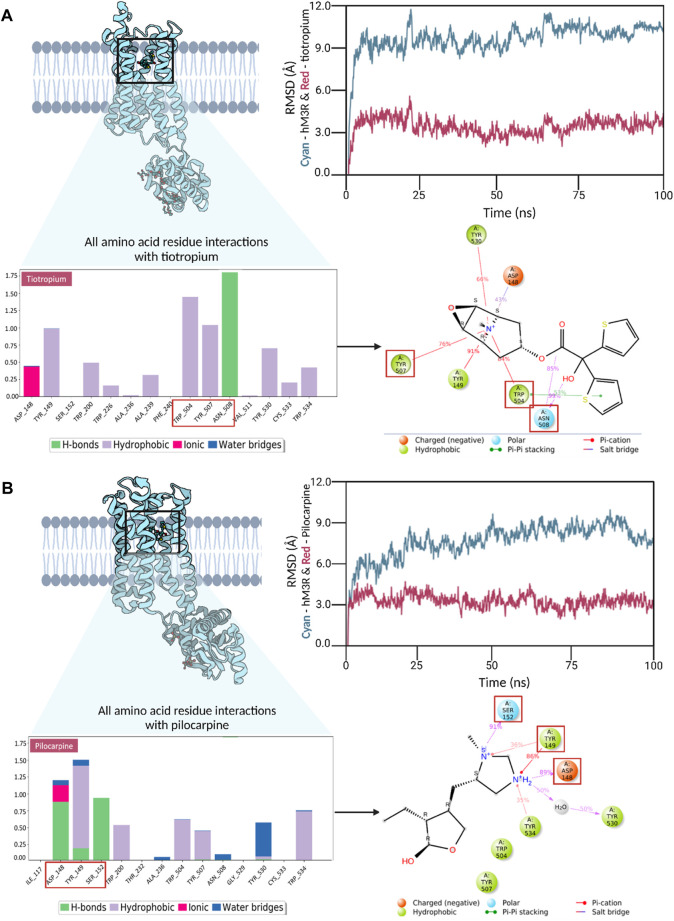
Trajectory analyses of molecular dynamics simulations for human M3R in complex with pilocarpine **(A)** and tiotropium **(B)**. Root-mean-square-deviation (RMSD) analysis demonstrated stable interactions in both the pilocarpine-hM3R and tiotropium-hM3R complexes, with pilocarpine displaying a tighter binding, likely due to its lower molecular mass. Interaction fraction analyses were conducted to reveal all amino acid residue interactions observed during MD simulation. The bar diagrams for pilocarpine and tiotropium revealed TYR149 as a key contributor in both complexes, engaging in diverse interactions with pilocarpine and tiotropium. Notably, TRP504 exhibited a significantly higher interaction fraction with tiotropium compared to pilocarpine. The top binding residues differed in their locations, with pilocarpine-hM3R interactions upstream (148-152) and tiotropium-hM3R interactions downstream (504-508), as denoted by red boxes. Subsequent 2D Ligand-protein contact analysis was conducted to visualize these key binding interactions that occurred more than 30% of the simulation time. Red squares in the 2D diagram correspond to top binding residues identified from the interaction fraction analysis. Overall, these results underscore the significance of a positively charged tertiary amine in the binding process to human M3R, hinting at the potential of aromatic side groups to amplify antagonistic properties, especially through interactions with TRP504.

To delve into additional interactions associated with human M3R agonism and antagonism, we conducted triplicate molecular dynamics simulations for human M3R docked with pilocarpine and tiotropium, respectively. Readers are encouraged to view the video analysis of the simulation results for the hM3R-pilocarpine and hM3R-tiotropium complexes (Supplementary Videos S1, S2). The root-mean-square-deviation (RMSD) analysis was performed for both complexes, indicating stability at a low RMSD along the timeframes (Figure 5). Towards the end of the simulation, pilocarpine achieved an RMSD of approximately 2.5 Å, while tiotropium stabilized at an RMSD of around 3.0 Å, signifying stable binding for both compounds.

Interaction fraction analyses of the pilocarpine-hM3R and tiotropium-hM3R complexes were also conducted. An interaction fraction value of 1.0 implies that the ligand contacted a binding residue throughout 100% of the simulation time. Values exceeding 1.0 indicate multiple binding forces. For instance, in the interaction fraction analysis of pilocarpine-hM3R, TYR149 exhibited an interaction fraction value of 1.498, engaging with pilocarpine through hydrogen bonds, hydrophobic forces (cation-π exchange), and water bridges. The top three contributors to the binding interaction for pilocarpine were TYR149, ASP148, and SER152 with scores of 1.498, 1.217, and 0.914, respectively. In contrast, the top three contributors to the binding interaction for tiotropium were ASN508, TRP504, and TYR507 with scores of 1.755, 1.477, and 1.083, respectively. Notably, TRP504 exhibited an interaction fraction value with tiotropium nearly three times higher (1.477) than with pilocarpine (0.591). Furthermore, the top three binding residues with pilocarpine were upstream at positions 148-152, whereas they were downstream at positions 504-508 for tiotropium. Ligand-protein contact analysis of pilocarpine-hM3R revealed ASP148, TYR149, and SER152 intimately associated with the nitrogen ring of pilocarpine. Meanwhile, the ligand-protein contact analysis of tiotropium-hM3R showcased the preservation of π-π stacking exchange via TRP504 during the entirety of the simulation, aligning with the results of molecular docking. Together, these findings emphasize the importance of a positively charged tertiary amine for binding to human M3R and suggest that aromatic side groups may enhance antagonistic properties, particularly through π-π interaction with TRP504.

Finally, for a comparative assessment of the protein-ligand complex stability between pilocarpine and tiotropium, the MM/GBSA method was employed to calculate the binding free energy (ΔG_bind_) (Supplementary Table S1). The MM/GBSA analysis indicated that the pilocarpine-hM3R and tiotropium-hM3R complexes exhibited predicted binding free energies of −46 kcal/mol and −96 kcal/mol, respectively. Remarkably, tiotropium demonstrated a more stable complex with hM3R than pilocarpine. Moreover, the nearly two-fold change in binding free energy suggests that once tiotropium binds to hM3R, its binding mode remains stable, and the transmembrane domain of human M3R containing the binding pocket likely maintains a stable, rigid conformation, aligning with the observations from the video analysis of the tiotropium-hM3R complex (Supplementary Video S2).

### 3.4 High-throughput virtual screening of compound libraries against human M3R homology model

**FIGURE 6:**
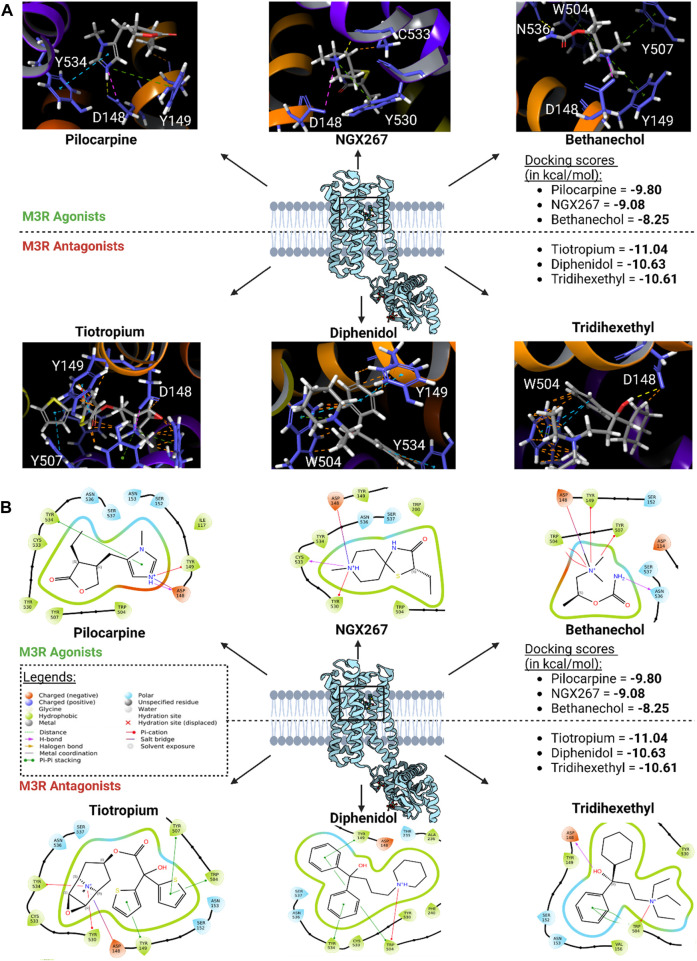
Top three scoring M3R agonists and antagonists upon molecular docking of six agonists and 15 antagonists of hM3R. **(A)** 3D binding modes of top three scoring M3R agonists and antagonists within human M3R binding site. Yellow dotted lines indicate hydrogen bond network; purple dotted lines salt bridges; green dotted lines cation-π interactions; blue dotted lines π-π stacking; and orange lines potential steric clash. Note that all three of them involve presence of extensive π-cation and salt bridge interactions with the nitrogen ring via ASP148, TYR149, TRP504, TYR507, and TYR530. **(B)** 2D visualization of relevant binding residues for the top three scoring M3R agonists and antagonists within human M3R binding site. In addition to the cation-π and salt bridge interactions found with the agonists, M3R antagonists all involved presence of extensive π-π stacking with TRP504, which may explain their improved docking scores compared to agonists.

The binding modes of the top three scoring known M3R agonists included the presence of cation-π exchange between a positively charged tertiary amine group and aromatic residues of human M3R, including TYR149, TRP504, and TYR507 (Figure 6). Additionally, hydrogen bonding between an oxygen group and ASN508 was another notable force. Similarly, the binding modes of the top three scoring known M3R antagonists exhibited cation-π exchange between a positively charged tertiary amine and residues TYR149, TRP504, and TYR507; ASN508 was also seen engaging through a hydrogen bond network. Notably, both M3R agonists and antagonists demonstrated a hydrogen bond network with ASN508, aligning with literature emphasizing the significance of this interaction across various muscarinic receptor subtypes (Kruse et al., 2013). However, in contrast to M3R agonists, the antagonists shared a consistent presence of π-π stacking exchange between their aromatic rings and TRP504 (Table 1). Notably, this interaction was absent in all tested hM3R agonists, as all of them lack aromatic side groups in their chemical structures except for pilocarpine. Meanwhile, 12 out of 15 M3R antagonists displayed π-π stacking exchange. Lastly, it is intriguing to observe that M3R antagonists overall achieved higher predicted affinities than agonists, likely attributed to the additional stability provided by π-π stacking exchange.

**TABLE 1:** High-throughput virtual screening of human M3R agonists (Casson, 2022) and antagonists (Goel et al., 2010).

HM3R agonists	Molecular Mass (g/mol)	Docking scores (kcal/mol)	π-π stacking with TRP504?	Functional groups
Pilocarpine	208.3	−9.839	No	Arene, tertiary amine, and lactone
NGX267	214.3	−9.082	No	Tertiary amine, carboxamide, carbonyl, sulfide
Bethanechol	161.2	−8.251	No	Ammonium, carbamate
Cevimeline	199.3	−8.108	No	Tertiary amine, ether, sulfide
Xanomeline	281.4	−6.019	No	Tertiary amine, ether, arene, alkene
Methacholine	160.2	−3.746	No	Ammonium, ester

HM3R agonists	Molecular Mass (g/mol)	Docking scores (kcal/mol)	π-π stacking with TRP504?	Functional groups
Tiotropium	392.5	−11.036	Yes	Arene, ammonium, ester, epoxide, sulfide
Diphenidol	309.4	−10.626	Yes	Arene, tertiary amine, ether
Tridihexethyl	318.5	−10.606	Yes	Arene, tertiary amine, hydroxyl
Methanetheline	340.4	−10.400	Yes	Arene, ammonium, ester
Solifenacin	362.5	−10.378	Yes	Arene, ammonium, ester
Glycopyrronium	318.4	−10.264	Yes	Arene, ammonium, ester
Oxybutynin	357.5	−10.159	Yes	Arene, ammonium, ester, alkyne
Oxyphencyclimine	344.4	−10.034	No	Arene, ester, amidine
Promethazine	284.4	−9.850	Yes	Aniline, tertiary amine, sulfide
Homatropine methylbromide	370.3	−9.79	No	Arene, ammonium, epoxide, ester
Procyclidine	287.4	−9.748	Yes	Arene, tertiary amine, benzene ring
Dicyclomine	309.5	−9.45	No	Tertiary amine, ester
Olanzapine	312.4	−9.443	Yes	Aniline, tertiary amine, sulfide
Ipratropium	332.5	−9.391	Yes	Arene, ammonium, ester
Tropicamide	284.4	−9.064	Yes	Arene, tertiary carboxamide, carbonyl

**FIGURE 7:**
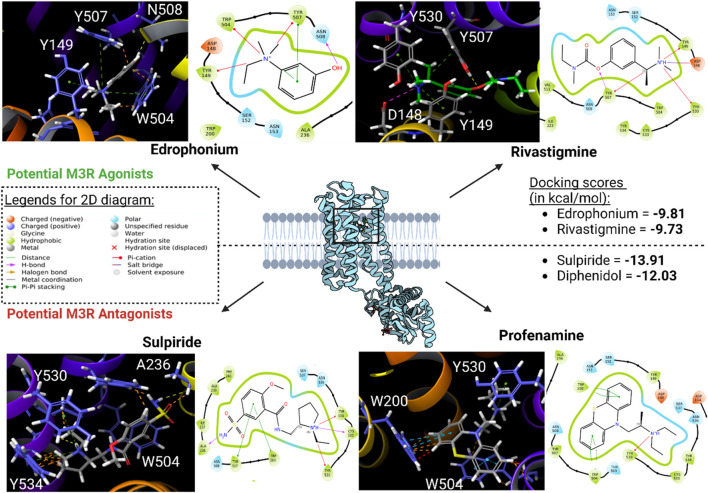
Potential human M3R agonists and antagonists upon high-throughput virtual screening of 1,667 FDA-approved or investigational drugs against human M3R downloaded from ZINC database. 3D analysis of binding interactions is accompanied by their associated 2D ligand interaction diagram for each hit compound. Recall that within the 3D analysis, yellow dotted lines indicate hydrogen bond network; purple dotted lines salt bridges; green dotted lines cation-π interactions; blue dotted lines π-π stacking; and orange lines potential steric clash. Note that both edrophonium and rivastigmine involve presence of cation-π with the tertiary nitrogen via TYR149, TRP504, and TYR530. Furthermore, their predicted affinities were comparable to the docking score of pilocarpine (−9.8 kcal/mol). Meanwhile, binding mode of potential antagonists of human M3R revealed the presence of π-π stacking with TRP504 in addition to hydrogen bonding and salt bridge interactions seen in M3R agonists.

Subsequently, high-throughput virtual screening was executed on human M3R, utilizing 1,667 FDA-approved or investigational drugs sourced from the ZINC database (Sterling and Irwin, 2015). After three rounds of molecular docking with Glide, the pool of drugs was narrowed down from 1,667 to four potential ligands for human M3R. The four lead compounds identified were edrophonium, rivastigmine, sulpiride, and profenamine (Figure 7). All four lead compounds exhibited extensive cation-π interactions involving their tertiary amine group and human M3R residues TYR149, TRP504, and TYR530. This aligns with our earlier findings, emphasizing the crucial role of the cation-π network in protein-ligand interactions, irrespective of agonism or antagonism. Notably, sulpiride and profenamine displayed additional π-π interactions between their aromatic rings and human M3R’s TRP504. Furthermore, sulpiride and profenamine demonstrated higher predicted affinities compared to edrophonium and rivastigmine. This is consistent with our previous observations that human M3R antagonists generally achieve higher predicted affinities than agonists, likely due to the added stability provided by π-π stacking exchange.

### 3.5 Assessment of lead compounds for IOP reduction in mice

**FIGURE 8:**
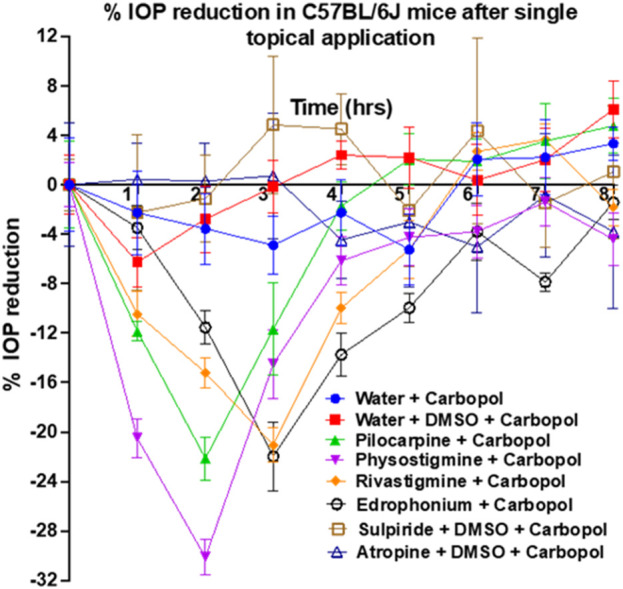
Assessment of pilocarpine, physostigmine, rivastigmine, edrophonium, sulpiride, and atropine for IOP reduction in mice. Pilocarpine and physostigmine reached their maximum IOP reduction at 2 h post-topical application. Notably, physostigmine exhibited an impressive 30% reduction in IOP, surpassing pilocarpine’s 22% reduction. Meanwhile, rivastigmine and edrophonium achieved approximately 21% IOP reduction, which is comparable to pilocarpine but less than physostigmine. The T_end_ of Rivastigmine was at 5 h, while that of edrophonium was at 5.5 h, suggesting longer durations of action than pilocarpine’s T_end_ of 4.4 h. Lastly, AUC values for rivastigmine and edrophonium exceeded that of pilocarpine, further suggesting prolonged action despite similar levels of IOP reduction among these three drugs. Diverse potency and toxicity profiles were observed among the investigated cholinergic drugs, suggesting that the cholinergic activation of the anterior segment of the eye for IOP reduction is more nuanced than previously understood.

Rivastigmine and edrophonium were formulated to assess their impact on IOP in mice after topical dosing (Figure 8). Our positive controls for IOP reduction, pilocarpine (a known agonist of human M3R) and physostigmine (a recognized acetylcholinesterase inhibitor) achieved peak IOP reduction approximately 2 h after topical application. Remarkably, physostigmine demonstrated a substantial 30% reduction in IOP, compared to pilocarpine’s 22% reduction (Table 2). However, physostigmine-treated mice exhibited significant cholinergic adverse effects, such as excessive salivation, urination, defecation, muscle paralysis, and hypoventilation. In contrast, mice treated with pilocarpine displayed only mild cholinergic adverse effects, like profuse salivation and urination. For our lead compounds, both rivastigmine and edrophonium achieved maximum IOP reduction approximately 2.8 h after topical application, with effects lasting longer than pilocarpine or physostigmine. The T_end_ of Rivastigmine was at 5 h, and that of edrophonium was at 5.5 h. Both compounds achieved roughly 21% reduction in IOP, comparable to pilocarpine but less than physostigmine. In terms of cholinergic adverse effects, mice treated with rivastigmine displayed similar effects to pilocarpine, notably excessive salivation, and urination. Intriguingly, mice treated with edrophonium exhibited virtually no adverse cholinergic side effects despite achieving a similar level of IOP reduction as pilocarpine and rivastigmine. It is also noteworthy that AUC values for rivastigmine and edrophonium were higher than pilocarpine, likely due to their longer duration of action, despite similar levels of maximum IOP reduction. Meanwhile, our negative control, atropine, had no impact on IOP reduction. Similarly, our anticipated M3R antagonist, sulpiride, did not induce IOP reduction. To our knowledge, this is the first study to showcase not only the capacity of rivastigmine and edrophonium to lower IOP in mice but also to underscore the diverse potency and toxicity profiles among cholinergic drugs upon topical application. Together, these findings emphasize that the cholinergic activation of the anterior segment of the eye for IOP reduction is more nuanced than previously perceived.

**TABLE 2:** Pharmacodynamic parameters after application of drops containing 0.4% pilocarpine, atropine, physostigmine, rivastigmine, and edrophonium to mouse eyes.

	Pilocarpine	Physostigmine	Rivastigmine	Edrophonium
T_max_ (h)	2.167 ± 0.376	1.917 ± 0.277	2.917 ± 0.515	2.833 ± 0.389
T_end_ (h)	4.417 ± 1.377	4.182 ± 0.751	5.000 ± 1.044	5.5 ± 1.567
% IOP reduction at T_max_	22.13 ± 4.251	30.13 ± 3.460	21.04 ± 3.471	21.960 ± 6.800
AUC (%^*^h)	56.27 + 11.47	82.93 + 10.03	66.15 + 6.679	73.03 + 8.672

### 3.6 Quantitative structure-activity relationships (QSAR) and ADME (absorption, distribution, metabolism, excretion) analysis of cholinergic drugs

Quantitative structure-activity relationships (QSAR) analysis was employed to predict the chemical properties of the investigated drugs at pH 7.4, corresponding to the pH of the tear film (Table 3). The calculation of cLogD_pH7.4_, representing the distribution coefficient of a drug at pH 7.4, indicated that rivastigmine exhibited the highest distribution coefficient, suggesting its predominantly hydrophobic nature at tear film pH. Conversely, edrophonium chloride demonstrated the lowest distribution coefficient, indicating its predominance as a hydrophilic compound at tear film pH. Subsequent analysis of ΔG_o/w_, representing the change in Gibbs free energy of drug distribution/partitioning, revealed that rivastigmine had the lowest free energy, aligning with its hydrophobic nature as suggested by its cLogD_pH7.4_. Conversely, edrophonium chloride exhibited the highest Gibbs free energy, consistent with its hydrophilic nature at pH 7.4 as indicated by its cLogD_pH7.4_. Meanwhile, sulpiride demonstrated the highest topological polar surface area (TPSA), while edrophonium chloride exhibited the lowest TPSA. Importantly, all tested drugs exhibited values of cLogD_pH7.4_, ΔG_o/w_, and TPSA that are favorable for crossing the corneal membrane, in accordance with Karami’s rule of thumb for optimal ocular permeability and absorption for topical eye drops (Karami et al., 2022). Notably, edrophonium chloride demonstrated the lowest distribution coefficient, highest Gibbs free energy of distribution/partitioning, and lowest TPSA among the tested drugs, all of which may be associated with its reduced cholinergic adverse effects observed in mice compared to rivastigmine, pilocarpine, and physostigmine.

**TABLE 3:** Quantitative structure-activity relationships (QSAR) analysis of cholinergic drugs and their physiochemical properties related to rule of thumb for optimal ocular permeability and absorption for topical eye drops proposed by Karami et al.

QSAR parameters	cLogD_pH7.4_	ΔG_o/w_ (kJ/mol)	TPSA (Å^2^)
Pilocarpine	1.39	−8.25	55.41
Physostigmine	1.24	−7.34	59.28
Rivastigmine	2.47	−14.6	41.24
Edrophonium Chloride	−1.90	11.28	23.12
Sulpiride	0.57	−3.37	110.91
Atropine	1.84	−10.91	59.81
Favorability for RO_x_^e^	<4.0	<20	<250

SWISS-ADME was employed to better understand the pharmacokinetics of the cholinergic drugs in relation to their biological activities observed in mice (Table 4). Pilocarpine exhibited moderate lipophilicity (LogP 1.13) and good solubility (LogS −1.38), enabling high gastrointestinal absorption and effective blood-brain barrier penetration without CYP450 interactions or P-glycoprotein substrate activity. Physostigmine demonstrated moderate lipophilicity (LogP 1.65) and lower solubility (LogS −2.64), but maintained high GI absorption and BBB permeability, with potential CYP2D6 inhibition and P-gp substrate status suggesting potential drug interactions and complex elimination route. Rivastigmine, with higher lipophilicity (LogP 2.34) and lower solubility (LogS −2.82), achieved high GI absorption and BBB permeability without CYP interactions or P-gp substrate involvement, indicating favorable pharmacokinetic properties. On the other hand, edrophonium was the only ligand to be characterized by low lipophilicity (LogP −1.07) and low-to-moderate solubility (LogS 0.28). It also had high GI absorption and BBB penetration without CYP interactions, but its P-gp substrate status may influence absorption and excretion. Sulpiride displayed low lipophilicity (LogP 0.83) and solubility (LogS −2.66) yet maintained high GI absorption. It is predicted to have low BBB permeability with no CYP interactions, though its P-gp substrate status may affect its absorption and elimination. Lastly, atropine, with higher lipophilicity (LogP 2.09) and lower solubility (LogS −2.70), also achieved high GI absorption and BBB permeability, but its CYP2D6 inhibition and P-gp substrate status suggest potential drug interactions and complex distribution.

**TABLE 4:** ADME (Absorption, Distribution, Metabolism, Excretion) Prediction and Analysis of Tested Cholinergic Drugs using SWISS-ADME (Daina et al., 2017). LogP represents the partition coefficient, indicating the compound’s lipophilicity and ability to cross cell membranes, while LogS denotes its aqueous solubility, impacting absorption and bioavailability. GI absorption assesses the probability of compound being absorbed through the gastrointestinal tract. CYP interactions highlight the compound’s interactions with cytochrome P450 enzymes, crucial for its metabolism. BBB permeability measures the compound’s ability to cross the blood-brain barrier, and P-gp substrate status identifies whether the compound is a substrate for P-glycoprotein, a transporter protein that can affect its absorption and excretion.

Tested drugs	LogP	LogS	GI absorption	CYP interactions	BBB permeability?	P-gp substrate?
Pilocarpine	1.13	−1.38	HIGH	NONE	YES	NO
Physostigmine	1.65	−2.64	HIGH	CYP2D6 Inhibition	YES	YES
Rivastigmine	2.34	−2.82	HIGH	NONE	YES	NO
Edrophonium	−1.07	0.28	HIGH	NONE	YES	YES
Sulpiride	0.83	−2.66	HIGH	NONE	NO	YES
Atropine	2.09	−2.70	HIGH	CYP2D6 Inhibition	YES	YES

## 4 Discussion

The correlation matrix analyses presented in this study shed light on the intricate relationship between muscarinic receptor mRNA expression and IOP dynamics within the anterior segment of the eye. Our findings revealed a prevailing negative correlation pattern between muscarinic receptor mRNA expression and IOP. Notably, this negative correlation reached statistical significance exclusively with *Chrm3* mRNA expression, corresponding to the M3 subtype of muscarinic receptor. The specificity of the negative correlation with *Chrm3* mRNA underscores the pivotal role of the M3 subtype in modulating IOP dynamics, providing further support for the targeted investigation of M3R in the context of IOP regulation. Furthermore, a negative correlation between *Chrm1* and IOP was observed, although it was not reported to be statistically significant (*p*-value = 0.0647). Note that the M1 receptor is the second most common muscarinic subtype expressed in the ciliary muscle and iris sphincter within the anterior segment of the eye (Gil et al., 1997; Nietgen et al., 1999). This suggests a compelling possibility that the M1 receptor may also play a role in regulating aqueous humor dynamics, alongside the M3 receptor. Another possibility is that *Chrm1* is known for its expression in the CNS, including the retina. Recall that the systems genetics analysis was conducted using BXD mice aged 1-30 months, with some subtypes, such as BXD50 and BXD51, known to develop glaucoma spontaneously (Pilkinton et al., 2023). Consequently, optic neuropathy resulting from elevated IOP may lead to reduced CNS expression of *Chrm1* in BXD mice and the subsequent negative correlation observed in systems genetics analysis of their whole eyes. In the meantime, an intriguing positive correlation pattern emerged when examining the relationship between *Chrm3* and the mRNA expressions of other muscarinic acetylcholine receptors. Together, these findings signify a potential multifaceted regulatory network among muscarinic receptor subtypes that could contribute to the modulation of IOP.

When we ran HTVS of muscarinic agonists and antagonists of M3R, we discovered none of the agonists displayed π-π stacking exchange and lacked the capacity to form one since they lack aromatic side groups. Similar to pilocarpine, all the other agonists shared presence of the cation-π exchange. There were more variations in the presence of hydrogen bond network with ASN508. For instance, NGX267 formed only one hydrogen bond with ASN508, as opposed to pilocarpine forming two, likely because NGX267 has only one oxygen molecule from the carbonyl group. Cevimeline did not form any interaction likely because its oxygen ring was oriented away from ASN508. Curiously, we discovered a unique interaction in the binding mode of Xanomeline that has an aromatic sulfur ring that forms π-π stacking exchange with TRP200. On the other hand, of the 15 antagonists we screened, 12 of them shared presence of π-π stacking exchange with TRP504. Among the three antagonists that did not share any π-π stacking exchange, dicyclomine was the only molecule that did not have an aromatic side group. Furthermore, among the top 10 high-affinity antagonists with docking scores < −10 kcal/mol, oxyphencyclimine was the only molecule containing an aromatic side group that did not form π-π stacking exchange with TRP504. Finally, it is important to address the limitation of molecular docking. Docking scores are mere estimates of the binding free energy of a protein-ligand complex. Ligand binding does not necessarily mean activation or inhibition, as protein activity is governed by a myriad of factors, such as temperature, pH, solvation, cofactor binding, and chaperones (Deller et al., 2016). Hence, molecular docking is a powerful tool to visualize protein-ligand interaction but may not be used to accurately predict experimental Ki or binding affinity (Ramirez and Caballero, 2016; Pantsar and Poso, 2018).

To assess M3R selectivity, we compared our findings with published structures of other muscarinic receptor subtypes bound to ligands. In human M1R, where HTL9936 serves as a partial agonist, cation-π exchange involving a tertiary amine and aromatic residues (TYR106, TRP378, and TYR381) was identified (PDB: 6ZG4) (Brown et al., 2021). This is analogous to the cation-π interactions observed between a ligand’s tertiary amine group and human M3R residues TYR149, TRP504, and TYR530. Hydrogen bonding with ASN382 in M1R mirrored the hydrogen bonding seen with ASN508 in human M3R. However, a notable divergence was the replacement of GLN110 in M1R by ASN152 in human M3R, allowing for a more expansive conformational space in the M3R binding pocket. In human M2R, an agonist interaction involves PHE182 (PDB: 4MQS) (Kruse et al., 2013). The equivalent phenylalanine residue in human M3R (PHE202) had a different orientation, allowing for a broader conformational space exploited in the design of the hM3R-selective small molecule, BS46 (Liu et al., 2018). Human M4R featured an aromatic cage formed by TYR113, TYR278, TYR416, TYR439, and TYR443, enhancing the rigidity of its binding pocket (PDB: 7TRK) (Thal et al., 2016). The binding mode of iperoxo, a super-agonist for human M4R, revealed cation-π interaction between the ligand’s tertiary amine group and TYR278 in human M4R, which is similar to that seen between a ligand’s tertiary amine group and human M3R’s TYR149 (Vuckovic et al., 2023). Lastly, the crystal structure of human M5R exhibited a substitution of GLY408 in human M3R by a charged residue, LYS528. Nevertheless, the binding mode of tiotropium in complex with human M5R closely parallels that of human M3R, featuring cation-π interaction with TYR417 as well as a double hydrogen bond network involving ASN395 and tiotropium’s hydroxyl and ester groups (PDB: 6OL9) (Vuckovic etal., 2019).

Our *in silico* assays highlighted the significance of a tertiary amine with a positively charged nitrogen molecule and the ability to hydrogen bond with ASN508 for M3R binding, irrespective of agonism or antagonism. This aligns with acetylcholine’s structure, an endogenous M3R ligand featuring a positively charged tertiary amine and a carboxylic acid group capable of hydrogen bonding with ASN508. For agonism promotion, it is advisable that the ligand lacks aromatic side groups and cannot engage in π-π stacking exchange with TRP504. These structural insights led to identifying two cholinergic drugs from a pool of 1,667 FDA-approved or investigational drugs demonstrating the most favorable profile for reducing IOP: edrophonium and rivastigmine. Edrophonium, an FDA-approved acetylcholinesterase inhibitor for myasthenia gravis diagnosis, cardiac arrhythmia management, and neuromuscular blocking agent toxicity reversal, induces mild cholinergic effects, including bradycardia and bronchoconstriction (Pakala et al., 2023). In the past, edrophonium was tested in myasthenia gravis and normal patients to assess for its impact on IOP reduction. However, the study delivered the drug through systemic injection, involved only five patients, and produced inconclusive results, thus limiting the clinical utility of the data (Kornblueth et al., 1960). Otherwise, as of February of 2024, there are no studies published in PubMed, Scopus, and Embase databases testing its efficacy on lowering IOP in any other experimental models. Rivastigmine, another FDA-approved acetylcholinesterase inhibitor for neurological symptoms in Alzheimer’s and Parkinson’s disease, triggers cholinergic hyperactivation-related adverse effects, like nausea and vomiting (Varadharajan et al., 2023). Significantly, the efficacy of reducing IOP using rivastigmine was examined in rabbit eyes via topical formulation, resulting in a 15.2% maximal IOP reduction with the application of 1% rivastigmine (Goldblum et al., 2000). However, the study was published as a conference paper with incomplete information on specific topical formulation utilized for the study and did not investigate systemic cholinergic adverse effects in rabbits. Otherwise, as of February of 2024, there are no studies published in PubMed, Scopus, and Embase databases demonstrating its efficacy on lowering IOP in any other experimental models.

Most notably, as of June 2023, the FDA withdrew its approvals of majority of acetylcholinesterase inhibitors, including both edrophonium and rivastigmine, in the US due to reported concerns over their adverse systemic effects, specifically QT prolongation and Torsade de pointes (Verdin, 2023). It is essential to highlight that despite these withdrawals, edrophonium and rivastigmine still possess the potential to indirectly reduce IOP by inhibiting acetylcholine degradation through acetylcholinesterase inhibition. Moreover, our *in silico* assays demonstrated high-affinity interactions, revealing that both edrophonium and rivastigmine favor agonist binding modes within the M3R binding site. Consequently, in the pursuit of repurposing existing cholinergic drugs, edrophonium and rivastigmine emerged as the most promising candidates for lowering IOP through manipulation of the parasympathetic nervous system of the eye. This discovery prompted a comparative analysis of their IOP-lowering effects and cholinergic toxicities in mice.

The outcomes of our *in vivo* investigation into the impact of topically administered rivastigmine and edrophonium on IOP highlighted their potential as safer cholinergic drugs for IOP reduction, surpassing the established FDA-approved pilocarpine. Both rivastigmine and edrophonium demonstrated a comparable level of IOP reduction to pilocarpine, yet their effects exhibited a prolonged duration. This extended efficacy suggests the possibility of reduced dosing frequency in glaucoma treatment, enhancing patient adherence and therapeutic outcomes. Pilocarpine IOP drops are associated with cholinergic adverse effects, including increased sweating, muscle tremors, blurry vision, and nausea/vomiting (Pakala et al., 2023; Panarese and Moshirfar, 2023). Despite efforts to develop topical formulations to mitigate its cholinergic adverse effects, the washout of drugs into the tear duct remains a challenge. This drainage route can lead to drug entry into the nasopharynx and eventually the gastrointestinal system, causing systemic side effects over time (Akhter et al., 2022). While rivastigmine displayed adverse cholinergic effects similar to pilocarpine, a remarkable observation was made with edrophonium, which exhibited virtually no cholinergic side effects. Recall that IOP study utilized a single topical drop of each drug and monitored IOP changes in the subsequent 8 h. Unfortunately, we did not prepare enough solution or chemical for a topical hydrogel formulation towards multiple doses over an extended period to assess their long-term systemic side effects. While there are studies exploring the systemic adverse effects of oral cholinergic drugs, there were scarce literature reporting systemic adverse effects associated with repeat doses of topical cholinergic drops. Since adverse cholinergic effects significantly constrain the clinical use of cholinergic drugs, it is imperative to conduct future longitudinal studies investigating the effects of multiple doses of these drugs, before we further test their IOP-reducing effects in other experimental models of glaucoma.

One potential limitation of our findings lies in the fact that both edrophonium and rivastigmine are acknowledged inhibitors of acetylcholinesterase. While our modeling studies suggest that these compounds favor M3R agonism, determining whether the observed IOP reduction is directly attributed to M3R agonism, indirectly through diminished acetylcholine degradation, or a combination of both is challenging. Notably, pilocarpine, our positive control, primarily reduces IOP through M3R agonism, while physostigmine, another positive control, achieves IOP reduction by inhibiting acetylcholinesterase. However, in animal studies, both edrophonium and rivastigmine exhibited pharmacodynamic profiles distinct from our positive controls, indicating a more nuanced mechanism beyond M3R agonism, acetylcholinesterase inhibition, or a combination of both. Additionally, sulpiride, a dopamine D2 receptor antagonist used in schizophrenia management, also inhibits acetylcholinesterase (Dross, 1977). Modeling studies revealed π-π stacking with TRP504, favoring M3R antagonism over agonism. Animal studies confirmed that sulpiride, like atropine (a known muscarinic antagonist), failed to reduce IOP in mice, suggesting two possibilities. The first possibility stems from the results of our modelling that sulpiride’s predicted M3R antagonism may have overridden the increased acetylcholine levels resulting from its inhibition of acetylcholinesterase. Secondly, sulpiride, known for its diverse off-target effects on P-glycoproteins, carbonic anhydrases, and ATP-binding cassette sub-family G member 2 (ABCG2), may introduce complexities confounding its role in IOP modulation (Sulpiride, 2006). Cumulatively, all these findings suggest that the cholinergic modulation of the anterior eye segment for IOP reduction is more intricate than previously understood. Consequently, our team conducted additional *in silico* assays on the investigated cholinergic drugs through QSAR and ADME analysis.

The QSAR results highlighted that edrophonium chloride demonstrated the smallest TPSA among the tested drugs. This characteristic is favorable for optimal corneal permeability, suggesting that a significant portion of topically applied edrophonium likely traversed the corneal membrane rather than being drained through the nasolacrimal duct into systemic circulation. This phenomenon could potentially account for its diminished systemic cholinergic adverse effects observed in mice upon topical application. Meanwhile, sulpiride was identified as having the highest TPSA, potentially leading to decreased corneal permeability, providing a third explanation for its inability to lower IOP when applied topically. Notably, both QSAR and ADME analyses identified edrophonium as the most water-soluble compound among the tested drugs. This offers another potential explanation to why mice treated with edrophonium exhibited reduced systemic cholinergic adverse effects compared to other drugs: its aqueous solubility facilitated its entrapment in the aqueous core of our Carbopol 981 hydrogel formulation, allowing for controlled release with reduced washouts through drainage routes on the ocular surface.

Together, our findings from docking, affinity studies, QSAR analysis, and ADME studies collectively support the significance of specific molecular features associated with cholinergic activation as well as their optimal pharmacokinetic properties. Docking and affinity studies demonstrated that a tertiary amine with a positively charged nitrogen and the ability to hydrogen bond with ASN508 are critical for M3R binding, irrespective of agonism or antagonism. These structural insights led to the identification of edrophonium and rivastigmine as promising drugs for reducing IOP among 1,667 FDA-approved or investigational drugs. *In vivo* studies confirmed their comparable efficacy in reducing IOP to pilocarpine, with edrophonium showing prolonged effects and minimal systemic cholinergic adverse effects. The QSAR analysis further highlighted edrophonium’s small TPSA as critical descriptor influencing drug permeability across biological membranes. This characteristic is advantageous for ocular drug delivery, enhancing local bioavailability while minimizing systemic absorption. Additionally, both QSAR and ADME analyses identified edrophonium as the most water-soluble compound among the drugs tested. Once delivered directly to the corneal epithelium, edrophonium’s solubility may contribute to its entrapment in the aqueous core of our hydrogel vehicle, allowing for sustained release and reduced entry to systemic circulation through drainage routes on the ocular surface. These findings collectively support the potential of edrophonium to guide the development of derivatives or new cholinergic drugs that can safely lower IOP in glaucoma with reduced cholinergic adverse effects.

Overall, our discoveries indicate that the cholinergic activation of the anterior eye segment for IOP modulation involves more complexity than simple acetylcholinesterase inhibition or M3 receptor agonism. These insights prompt a re-evaluation of the parasympathetic system in the anterior eye segment for the development of safe and effective treatments for glaucoma. In terms of clinical implications, the combination of reduced systemic side effects and an extended duration of action positions edrophonium and its derivatives as attractive candidates for further investigation in experimental animal models of glaucoma.

## 5 Conclusion

In conclusion, our investigation into the molecular interactions, binding affinities, and *in vivo* effects of cholinergic drugs, specifically rivastigmine and edrophonium, has provided valuable insights into their potentials for IOP reduction with distinct pharmacokinetic profiles when compared to pilocarpine. While edrophonium has demonstrated potential as an IOP-reducing agent with fewer cholinergic adverse effects in mice, it is imperative to recognize its discontinuation in the United States and the reported adverse effects in patients that prompted this decision. Keeping this in mind, future research endeavors can utilize these insights to facilitate the clinical translation of edrophonium’s IOP-lowering effects, potentially leading to the development of its derivatives that may expand therapeutic options against glaucoma. This also underscores the potential need for re-opening the investigation of the cholinergic system in the eye, aiming to develop novel drugs capable of lowering IOP with a safer toxicity profile.

## Data Availability

The original contributions presented in the study are included in the article/Supplementary Material, further inquiries can be directed to the corresponding author.

## Supplementary material

SUPPLEMENTARY FIGURE S1
The full amino acid sequence of human M3 muscarinic receptor (M3R) is presented with its major tertiary domains color-coded for clarity. Molecular weight (MW) and isoelectric point (pI) were calculated from this sequence using the SWISS-EXPASY pI/MW tool. It is important to note that the homology model used in our studies lacks intracellular loop 3 (indicated by a green box), which is absent from the structural representation. However, this omission is not expected to influence the interaction dynamics at the binding site, primarily located within the transmembrane domain.

SUPPLEMENTARY FIGURE S2
Comparison of the binding mode of Iperexo (a muscarinic super-agonist) and that of pilocarpine. 3D binding modes of iperexo, pilocarpine, and their overlay within human M3R binding site were presented on the top row. Yellow dotted lines indicate hydrogen bond network; purple dotted lines salt bridges; green dotted lines cation-π interactions. Note that both iperexo and pilocarpine have similar conformations with their positively charged tertiary amine oriented towards ASP148 and their aromatic rings towards VAL156 at the other end. 2D protein-ligand interaction diagrams of iperexo and pilocarpine were presented on the bottom row. The binding modes of both compounds involved presence of extensive π-cation and salt bridge interactions with the nitrogen ring via ASP148, TYR149, and TYR507. Also note the presence of hydrogen bonding between pilocarpine’s carbonyl ring and human M3R’s TRP200. There was no sign of π-π stacking interaction with TRP504.

SUPPLEMENTARY FIGURE S3
Ramachandran plot analysis of template M3R (PDB: 8E9Z) in comparison to human M3R homology model. Each dot on the plot represents a peptide with corresponding phi-psi angles. Blue dots indicate “allowable” peptides with favorable phi-psi angles, while red dots indicate “disallowed” peptides due to unfavorable phi-psi angles. Peptides with phi/psi angles around -90/120 degrees correspond to beta sheets, -90/-30 degrees to right-handed alpha helices, and 90/60 degrees to left-handed alpha helices. Overall, the analysis revealed a similar structural motif between the template and the homology model. The template had only 2 “disallowed” peptides, compared to 30 “disallowed” peptides in the homology model.

SUPPLEMENTARY VIDEO S1
Video analysis of the 100 ns MD simulation result for hM3R-pilocarpine complex, where hM3R is depicted as green cartoon structure and pilocarpine as cyan stick structure.

SUPPLEMENTARY VIDEO S2
Video analysis of the 100 ns MD simulation result for hM3R-tiotropium complex, where hM3R is depicted as orange cartoon structure and tiotropium as cyan stick structure.

SUPPLEMENTARY TABLE S1
MM/GBSA binding free energy calculation of hM3R-pilocarpine and hM3R-tiotropium complexes (N = 3). All values are in kcal/mol. Standard deviations less than 0.01 kcal/mol are not depicted.
