# Corrigendum: Deepening insights into cholinergic agents for intraocular pressure reduction: systems genetics, molecular modeling, and *in vivo* perspectives

**DOI:** 10.3389/fmolb.2024.1490100

**Published:** 2024-10-04

**Authors:** Minjae J. Kim, Mohamed M. Ibrahim, Monica M. Jablonski

**Affiliations:** ^1^ Department of Ophthalmology, The Hamilton Eye Institute, The University of Tennessee Health Science Center, Memphis, TN, United States; ^2^ Department of Pharmaceutics, Faculty of Pharmacy, Mansoura University, Mansoura, Egypt; ^3^ Department of Pharmaceutical Sciences, University of Tennessee Health Science Center, Memphis, TN, United States

**Keywords:** glaucoma, parasympathetic nervous system, systems genetics, virtual screening, molecular dynamics, quantitative structure-activity relationship, and *in vivo* efficacy

In the published article, there was an error in the **Acknowledgments**. The statement was incomplete and did not mention Robert Williams and Lu Lu. The correct **Acknowledgements** statement appears below.

“We extend our gratitude to Kirk Hevener and Wei Li for providing access to the molecular modeling station at the College of Pharmacy, University of Tennessee Health Science Center. We also extend our gratitude to Robert Williams and Lu Lu at the Department of Genetics, Genomics and Informatics, University of Tennessee Health Science Center. Funding was provided by a Challenge Grant from Research to Prevent Blindness to the Hamilton Eye Institute and R01EY021200 to MJ. **Figures 1–4** were generated using BioRender.”

The authors apologize for this error and state that this does not change the scientific conclusions of the article in any way. The original article has been updated.

