# Supplementary figures and images for: Deepening insights into cholinergic agents for intraocular pressure reduction: systems genetics, molecular modeling, and in vivo perspectives
Source: Front Mol Biosci. 2024 Jul 26;11:1423351. doi: 10.3389/fmolb.2024.1423351 (PMC11310038; doi:10.3389/fmolb.2024.1423351)

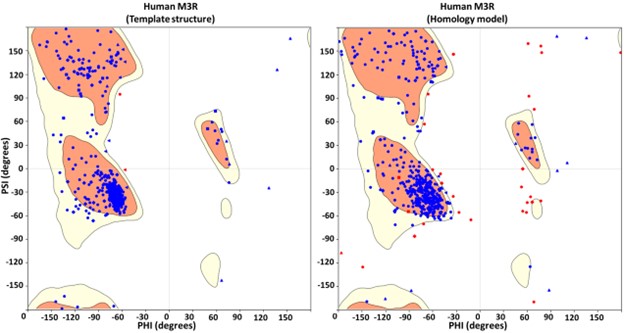

Supplement: Supplementary file 2 [file Image3.jpeg]

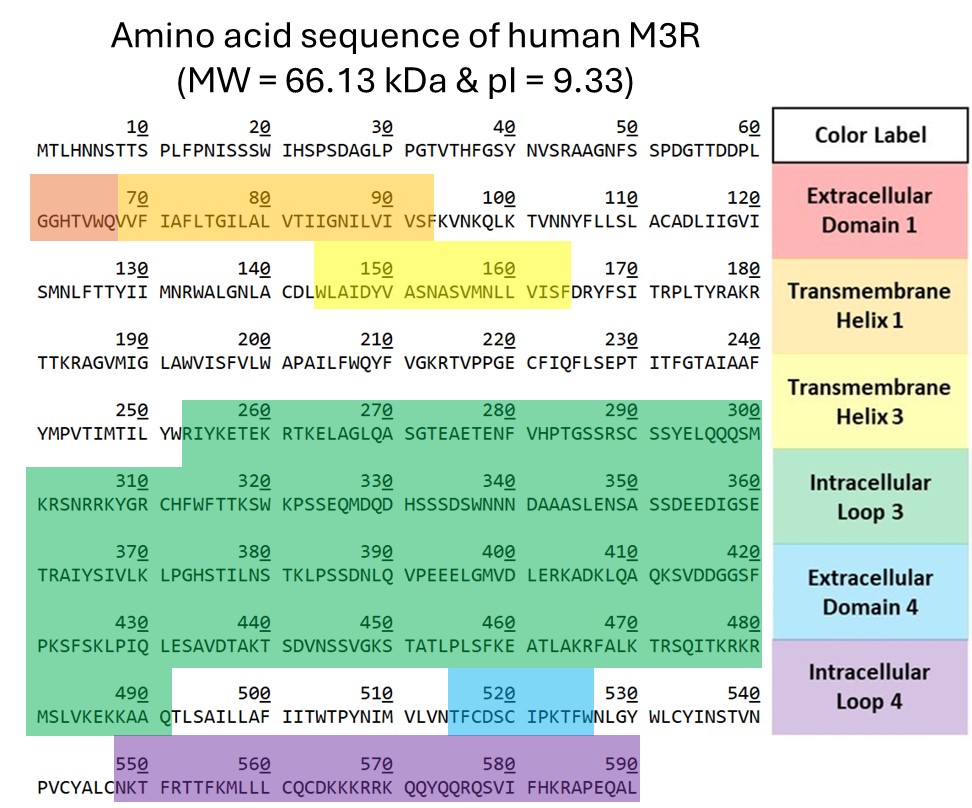

Supplement: Supplementary file 5 [file Image1.jpeg]

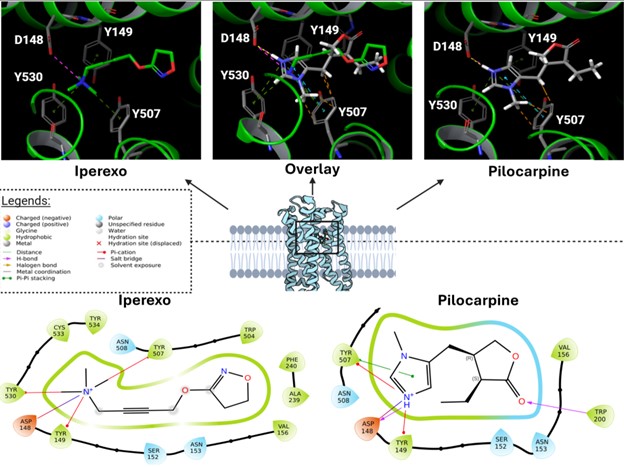

Supplement: Supplementary file 6 [file Image2.JPEG]
