# Supplementary material for: Deepening insights into cholinergic agents for intraocular pressure reduction: systems genetics, molecular modeling, and in vivo perspectives
Source: Front Mol Biosci. 2024 Jul 26;11:1423351. doi: 10.3389/fmolb.2024.1423351 (PMC11310038; doi:10.3389/fmolb.2024.1423351)
Supplement: Supplementary file 4 [file Table1.DOCX]

Supplemental Table 1. MM/GBSA binding free energy calculation of hM3R-pilocarpine and hM3R-tiotropium complexes (N = 3). All values are in kcal/mol. Standard deviations less than 0.01 kcal/mol are not depicted.

| **Protein-Ligand Complex​** | **ΔG_coulomb_​** | **ΔG_vdW_​** | **ΔG_covalent_​** | **ΔG_solv_​** | **ΔG_self-contact_​** | **ΔG_H-bond_​** | **ΔG_Lipo_​** | **ΔG_Packing_​** | **ΔG_Bind_​** |
| --- | --- | --- | --- | --- | --- | --- | --- | --- | --- |
| Human M3R-  Pilocarpines | -0.99 | -37.43  + 0.05 | 0.89​ | 12.94​  + 0.02 | 0​ | -1.82 | -20.39  + 0.03 | 0​ | -46.81 |
| Human M3R-  Tiotropium | 4.73​ | -60.93  + 0.04 | 2.72​ | -2.74 | 0​ | -1.22 | -36.04  + 0.02 | -3.34​ | -96.82 |
